# LRP-1 Promotes Cancer Cell Invasion by Supporting ERK and Inhibiting JNK Signaling Pathways

**DOI:** 10.1371/journal.pone.0011584

**Published:** 2010-07-14

**Authors:** Benoit Langlois, Gwenn Perrot, Christophe Schneider, Patrick Henriet, Hervé Emonard, Laurent Martiny, Stéphane Dedieu

**Affiliations:** 1 Université de Reims Champagne-Ardenne, CNRS UMR 6237 MEDyC, Laboratoire SiRMa, Campus Moulin de la Housse, Reims, France; 2 Cell Biology Unit, de Duve Institute and Université Catholique de Louvain, Brussels, Belgium; Calypte Biomedical Corporation, United States of America

## Abstract

**Background:**

The low-density lipoprotein receptor-related protein-1 (LRP-1) is an endocytic receptor mediating the clearance of various extracellular molecules involved in the dissemination of cancer cells. LRP-1 thus appeared as an attractive receptor for targeting the invasive behavior of malignant cells. However, recent results suggest that LRP-1 may facilitate the development and growth of cancer metastases in vivo, but the precise contribution of the receptor during cancer progression remains to be elucidated. The lack of mechanistic insights into the intracellular signaling networks downstream of LRP-1 has prevented the understanding of its contribution towards cancer.

**Methodology/Principal Findings:**

Through a short-hairpin RNA-mediated silencing approach, we identified LRP-1 as a main regulator of ERK and JNK signaling in a tumor cell context. Co-immunoprecipitation experiments revealed that LRP-1 constitutes an intracellular docking site for MAPK containing complexes. By using pharmacological agents, constitutively active and dominant-negative kinases, we demonstrated that LRP-1 maintains malignant cells in an adhesive state that is favorable for invasion by activating ERK and inhibiting JNK. We further demonstrated that the LRP-1-dependent regulation of MAPK signaling organizes the cytoskeletal architecture and mediates adhesive complex turnover in cancer cells. Moreover, we found that LRP-1 is tethered to the actin network and to focal adhesion sites and controls ERK and JNK targeting to talin-rich structures.

**Conclusions:**

We identified ERK and JNK as the main molecular relays by which LRP-1 regulates focal adhesion disassembly of malignant cells to support invasion.

## Introduction

The low-density lipoprotein (LDL) receptor-related protein-1 (LRP-1) is a ubiquitously expressed endocytic receptor belonging to the LDL-receptor family [1]. First described as a cargo receptor mediating the uptake and lysosomal degradation of α2-macroglobulin [2], LRP-1 was then found to be involved in the internalization of over 30 functionally and structurally unrelated extracellular ligands. These include proteases, protease-inhibitor complexes, macromolecular proteins and growth factors. Initially synthesized as a 600 kDa precursor, LRP-1 is further processed in the trans-Golgi by a furin-convertase for expression at the cell surface in the mature two-chain form composed of a 515 kDa extracellular subunit (α-chain), noncovalently linked to a 85 kDa β-chain containing the transmembrane domain and cytoplasmic tail. The LRP-1 α-chain harbors four ligand-binding clusters involved in the specific recognition of extracellular ligands and the assembly of multiprotein complexes at the cell surface. The intracellular domain of the LRP-1 β-chain could recruit molecules involved in the endocytic machinery and cytoplasmic modulators of signaling pathways [3].

The diversity of its ligands may explain why LRP-1 has been identified as a critical factor in diverse pathological contexts including atherosclerosis and neurodegenerative disorders as the most frequently described [4], [5]. A growing number of evidence strengthened the putative role of LRP-1 in crucial events during cancer progression [6]. LRP-1 was indeed reported to mediate the clearance of various matrix metalloproteinases such as MMP-2, MMP-9 and MMP-13 [7], [8], [9] and to regulate the plasmin activation cascade through endocytosis of tissue-type (tPA) or urokinase-type (uPA) plasminogen activators [10], [11]. Considering its well-known function in the control of matrix proteolysis [12], LRP-1 was initially proposed as a novel tumor suppressor. The weak expression level of LRP-1 observed in high grade human cancer cells and tissues seemed to support such a hypothesis [13], [14]. However, the overall function of LRP-1 in carcinogenesis appears to be much more complex than first thought. Recent studies have reported a positive contribution of LRP-1 to migration and invasion events of various cell types [10], [15], [16], including malignant tumor cells [17], [18], [19], [20]. LRP-1 expression was also reported to be hypoxia-responsive and to support the metastatic dissemination of mouse tumor xenografts [21]. Furthermore, LRP-1 was shown to sustain the mitogenic and/or promigratory effects of several soluble factors present in the peritumoral environment, thus supporting a pro-tumorigenesis role of the receptor [15], [22], [23]. We recently demonstrated that LRP-1 contributes to carcinoma cell invasion by subtly controlling adhesive complex turnover [17]. It therefore appears that the mechanisms by which LRP-1 controls tumor progression are not solely related to its endocytic function.

Beyond endocytosis, LRP-1 was distinguished by its ability to trigger intracellular signaling pathways regulating cell proliferation, differentiation, migration or survival [15], [24], [25], [26]. Its short intracytoplasmic domain (ICD) contains two NPxY motifs for phosphorylation by tyrosine kinases which are then able to bind phosphotyrosine-binding domain (PTB)-containing proteins. Yeast two-hybrid assays and proteomics analysis revealed that Shc (Src homology 2 domain containing protein), Fe65, Dab1 (disabled 1), PI3K (phosphatidyl-inositol 3-kinase) or a PIP-4,5-kinase (phosphatidyl-inositol-4,5-kinase) homologue may associate with the LRP-1-ICD [27], [28]. Thus, in response to extracellular stimuli, LRP-1 can recruit intracellular scaffold proteins to trigger downstream signaling. LRP-1 has been identified as a molecular signaling partner for platelet-derived growth factor receptor (PDGFR), leading to migratory and proliferative signaling and development of atherosclerotic lesions [4], [29]. More recently, the promigratory effect of the plasminogen activator inhibitor PAI-1 appeared to require LRP-1-dependent activation of the JAK (janus kinase)/STAT (signal transducer and activator of transcription) signaling pathway [30]. Furthermore, Ma and colleagues reported that LRP-1 might regulate murine embryonic fibroblasts migration by suppressing the Rac1 and extracellular signal-regulated kinase (ERK) pathways [31].

In the cancer field, there is a notable lack of knowledge about intracellular signaling downstream of LRP-1 and understanding of its possible contribution to cancer progression. In the present study, we characterized the molecular signaling relays involved in the LRP-1-mediated stimulation of cancer cell invasion and identified the LRP-1 β-chain as a main docking site for focal adhesion (FA) components and mitogen-activated protein kinase (MAPK)-containing complexes.

## Results

### Identification of LRP-1 as a regulator of MAPK signaling pathways in tumor cell context

**Figure 1:**
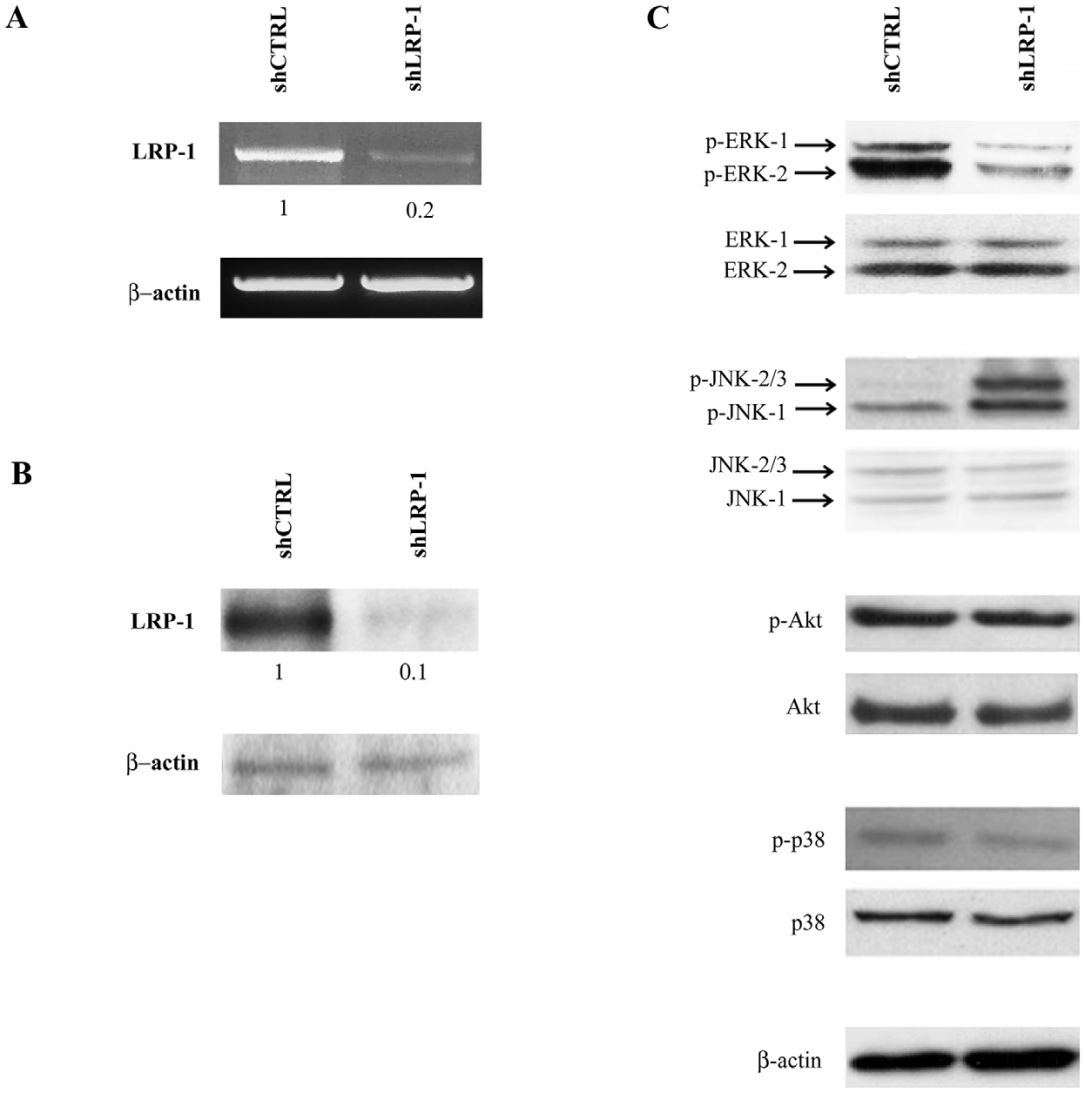
The shRNA-mediated silencing of LRP-1 inhibits ERK and triggers the activation of JNK signaling pathways. (**A**) Total RNAs were purified from FTC133 control clonal cell line (shCTRL) or clonal cells that stably overexpress shRNAs for LRP-1 (shLRP-1). The transcriptional level of LRP-1 was assessed by RT-PCR. β-actin primers were used as a normalization control. (**B**) Whole-cell extracts from each cell line were subjected to immunoblot analysis with anti-LRP-1 β-chain antibody (5A6). β-actin antibody was used for normalization. (**C**) shCTRL and shLRP-1 clonal cells were cultivated for 24 hours on gelatin-coated surfaces in 10% FBS-containing media. Whole-cell extracts were immunoblotted by using phospho-ERK, phospho-JNK, phospho-Akt and phospho-p38 antibodies. Antibodies to ERK, JNK, p38 and β-actin were used to ensure equal loading and for normalization. The gel and immunoblots presented are representative of at least three seperate experiments. Numbers under the gel and immunolots indicate the fold inductions by comparaison with shCTRL cells.

To assess the role of LRP-1 in the regulation of intracellular signal transduction, we used a previously validated method to generate shLRP-1 clonal cells that stably overexpress a specific hairpin sequence directed against LRP-1 [17]. As shown in Fig. 1, we observed a ∼90% down-regulation of endogenous LRP-1 expression in shLRP-1 cells compared with shCTRL cells, at both mRNA (Fig. 1A) and protein levels (Fig. 1B). We therefore analyzed the effects of LRP1 silencing on the activation of several potential LRP-1-regulated signaling pathways. The activation states of two major MAPK pathways, i.e., ERK-1/2 and SAPK/JNK (stress-activated protein kinase/c-jun N-terminal kinase), were first examined (Fig. 1C). We found that the level of phosphorylated ERK-1/2 was selectively decreased in LRP1-silenced cells and an increase in JNK-1/2/3 phosphorylation was detected upon LRP-1 silencing. However, the phosphorylation levels of Akt and p38 MAPK did not change significantly in LRP-1-deficient carcinomas. These results demonstrate that LRP-1 may act as an intracellular signaling modulator, activating ERK and inhibiting JNK signaling pathways.

### LRP-1 mediates the serum-induced activation of ERK-1/2 and constitutive inhibition of JNK-1/2/3

**Figure 2:**
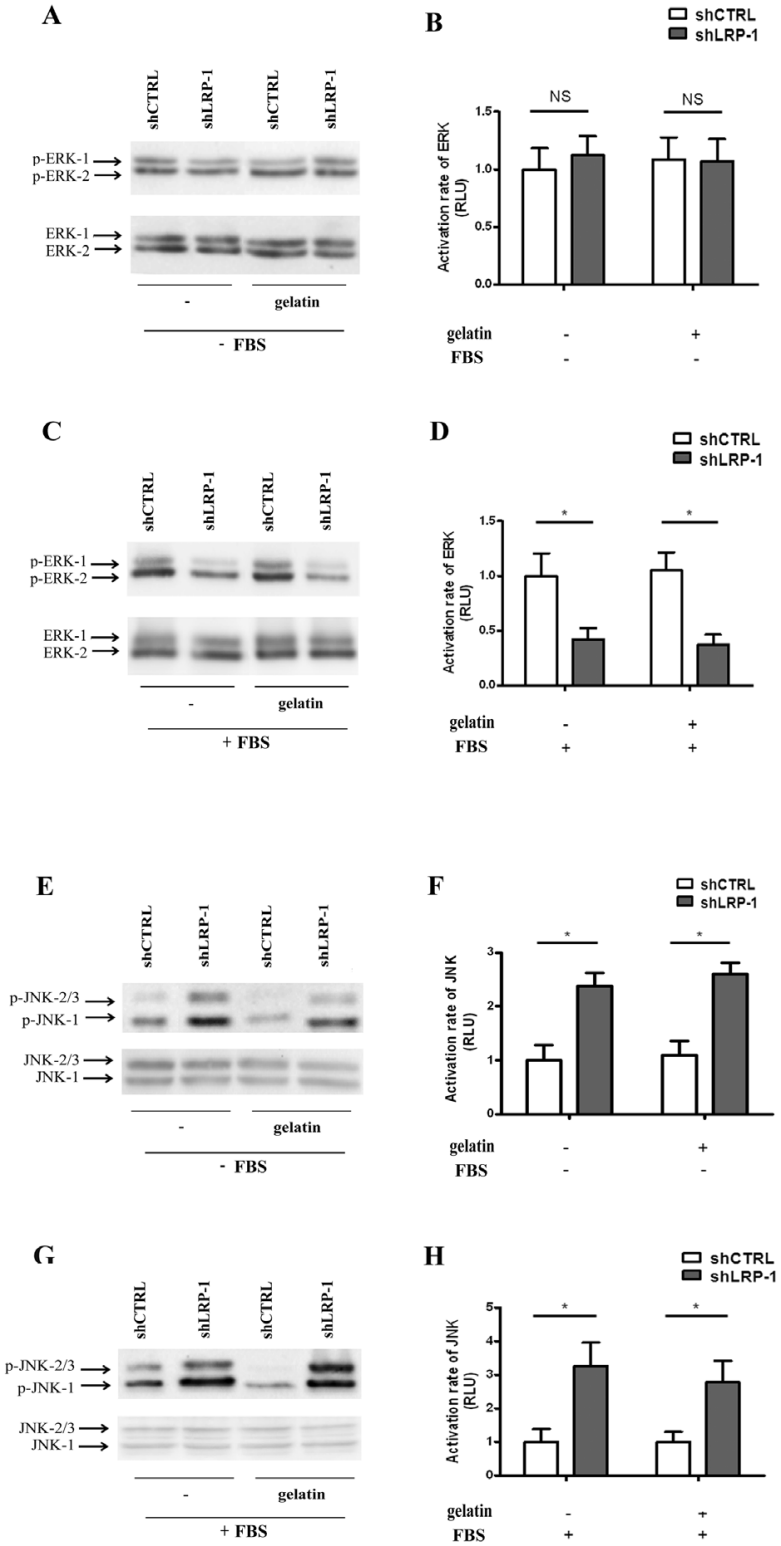
LRP-1 silencing inhibits the serum-mediated activation of ERK and leads to JNK hyperactivation. shCTRL and shLRP-1 cells were plated on plastic or gelatin-coated dishes for 24 hours in the absence or presence of FBS. Whole-cell extracts were subjected to Western-blot analysis to study the activation of ERK (**A–D**) and JNK (**E–H**) in the absence or presence of FBS. The respective activation rates of ERK (**B, D**) and JNK (**F, H**) were determined as intensity ratios of phospho-protein to corresponding pan-protein and expressed in relative units +/− SD, with a value of 1 ascribed to shCTRL cells. NS, not significant; *, *P*<0.05.

The LRP-1-mediated regulation of intracellular signaling can either depend on the binding of extracellular ligands or on the recruitment of intracellular scaffolds. We assessed LRP-1-mediated regulation of both ERK (Fig. 2A to 2D) and JNK pathways (Fig. 2E to 2H) in response to serum stimulation and gelatin coating. The LRP-1-mediated activation of ERK-1/2 phosphorylation was observed only in the presence of serum (Fig. 2A and 2B vs 2C and 2D). In contrast, the activation of JNK-1/2/3 in LRP-1-silenced cells was not conditioned by the presence of serum (Fig. 2E to 2H). In both cases, the regulation of MAPK by LRP-1 was unaffected by gelatin coating (Fig. 2A, 2C, 2E and 2G). The LRP-1 receptor therefore triggers the extracellular-ligand-dependent activation of ERK-1/2 and acts as a constitutive inhibitor of the JNK pathway.

### LRP-1 constitutes a docking site for Src, ERK and JNK containing complexes

**Figure 3:**
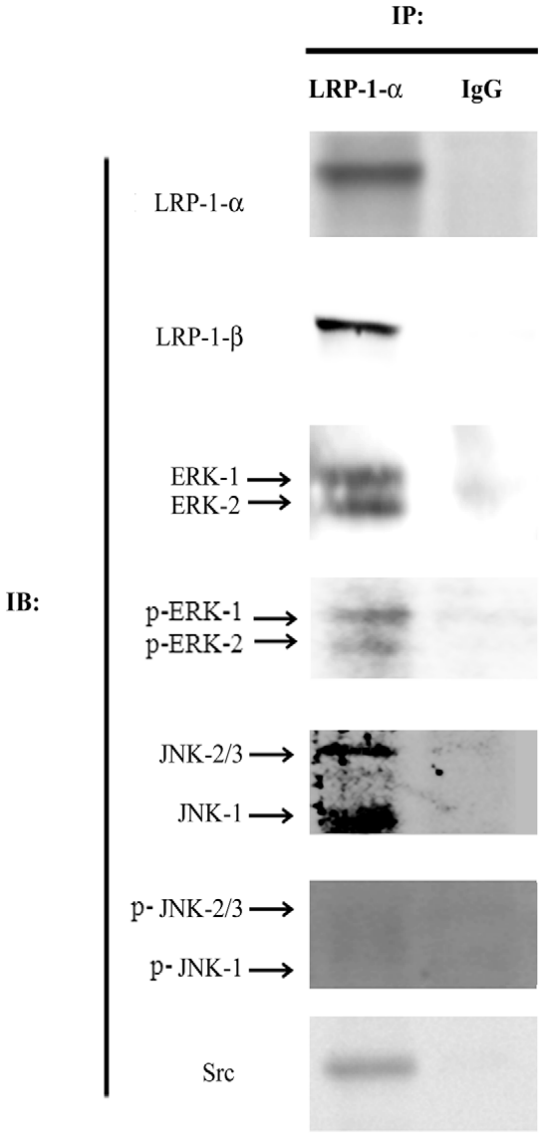
ERK, JNK and Src are co-immunoprecipitated with LRP-1 in tumor cells. Immunoprecipitations of LRP-1-containing complexes (IP: LRP-1-α) were performed by using anti-LRP-1 α-chain (8G1) antibodies and the immunocomplexes were immunoblotted (IB) by using 8G1, anti-LRP-1 β-chain (5A6), anti-ERK-1/2, anti-phospho-ERK-1/2, anti-JNK-1/2/3, anti-phospho-JNK-1/2/3 and anti-Src antibodies. Nonspecific IgGs were used as a negative control of immunoprecipitation.

The LRP-1 C-terminal end may interact with scaffold molecules involved in signal transduction [28]. Coimmunoprecipitation experiments were performed to test whether the intracellular domain of LRP-1 is able to recruit targets involved in the regulation of ERK and JNK signaling pathways in a tumor cell context. As shown in Fig. 3, we were able to successfully immunoprecipitate LRP-1 β-chain with an antibody raised against the extracellular LRP-1 α-chain. Both ERK and JNK kinases were co-immunoprecipitated with LRP-1 β-chain. We also examined the phosphorylation state of these kinases. Phosphorylated forms of ERK-1/2 were found associated with LRP-1, where phosphorylated JNK were not. Src, a kinase well-known to be activated downstream of mitogen and matrix receptors, was also detected in LRP-1 β-chain containing complexes.

### LRP-1-dependent ERK activation contributes to carcinoma cell invasion

**Figure 4:**
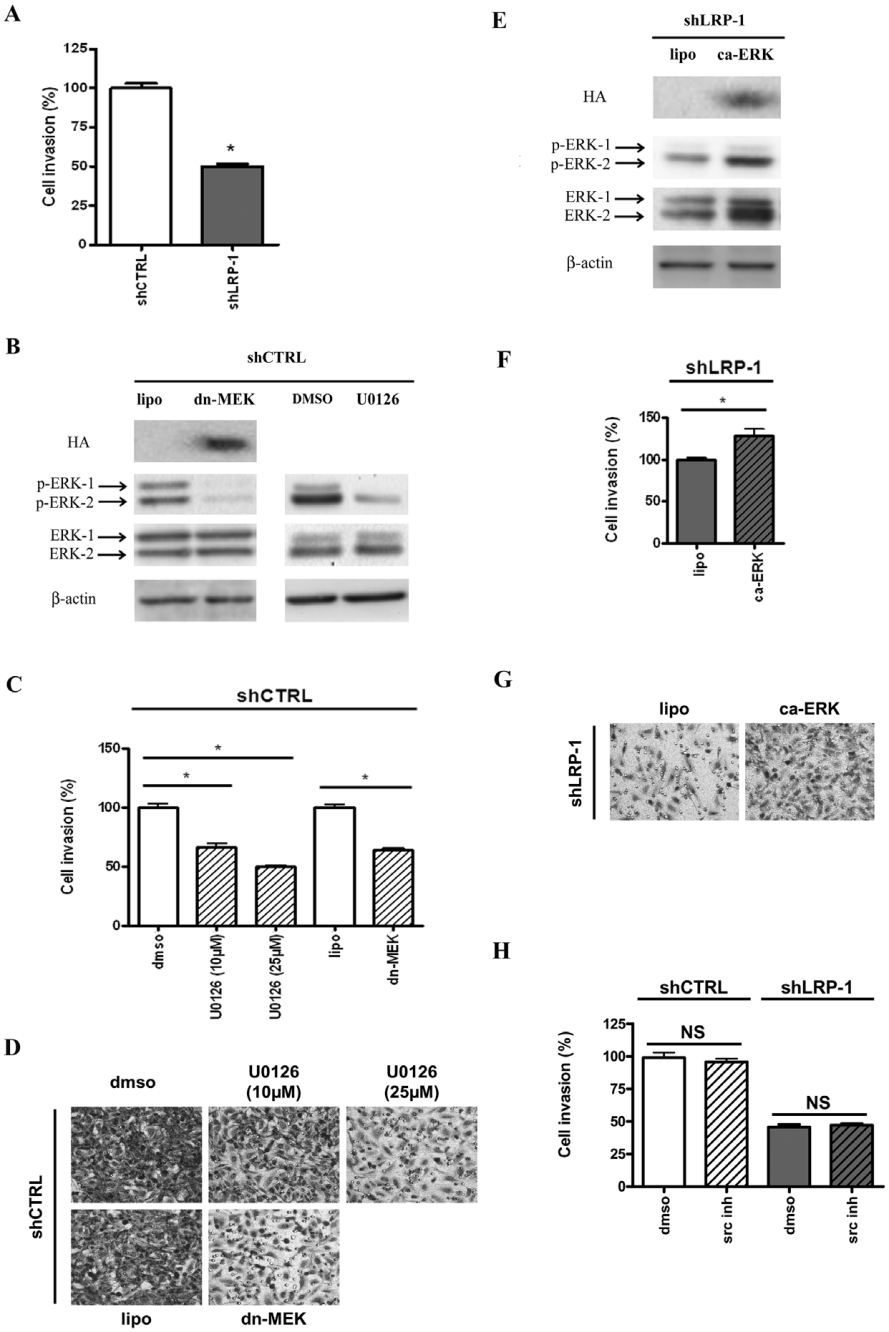
LRP-1 controls carcinoma cell invasion through a Src-independent activation of ERK. Matrigel Invasion assay was carried out for shCTRL and shLRP-1 carcinoma cells in basal conditions (**A**), following the modulation of ERK activity in shCTRL (**B–D**) and shLRP-1 cells (**E–G**) or in the presence of Src inhibitor (**H**). (**C, D**) The capacity of shCTRL cells to invade matrigel was quantified after inhibition of ERK activity by using U0126 treatment (10 or 25 µM) or expression of a kinase-dead mutant for MEK-1 (dn-MEK). (**F, G**) The invasive properties of shLRP-1 cells were examined after a constitutive activation of ERK-2 (ca-ERK). The efficiency of the ERK inhibition in shCTRL (**B**) and ERK activation in shLRP-1 cells (**E**) was controlled by using phospho-ERK, ERK and β-actin antibodies. Anti-HA was used to control the level of expression of overexpressed HA-tagged proteins. (**H**) Tumor cell invasion was measured after inhibition of Src-dependent activity by using 10 µM of Src kinase inhibitor I (Src inh). Representative images are shown for each condition (**D, G**). Results were obtained from three separate experiments each performed in triplicate. Invasion was determined by counting cells in eight random microscopic fields per well. Results were expressed as means +/- SD after normalization by comparison with vehicle, i.e., DMSO for drug treatments or lipofectamine (lipo) for transfection. NS, differences with corresponding control were not significant. *, *P*<0.05.

We recently described a key role for LRP-1 in tumor progression [17]. Indeed, as shown in Fig. 4A, LRP-1-deficient cells exhibited a two-fold decreased invasive capacity, as compared to control clonal cells. We therefore investigated whether the LRP-1-dependent activation of ERK could contribute to carcinoma cell invasion. First, control cells were treated with U0126, a selective inhibitor of MEK-1/2 (MAPK ERK kinase-1/2) or transfected with a dominant-negative mutant of MEK-1. The efficiency of ERK-1/2 inhibition under these conditions can be seen in Fig. 4B. No change in JNK phosphorylation was detected upon ERK inhibition (Fig. S1). Control cell invasion was inhibited upon U0126 treatment in a dose-dependent manner (Fig. 4C and 4D). Moreover, shCTRL cells overexpressing a kinase-dead form of MEK-1 exhibited reduced invasive properties (Fig. 4C and 4D). These results indicate that the ERK signaling module is activated during carcinoma cell invasion. Secondly, to rescue the activated ERK pathway in LRP-1-silenced cells, a constitutively-active ERK-2 was overexpressed. The activation state of ERK-1/2 was assessed by immunoblotting (Fig. 4E). As shown in Fig. 4F and 4G, the ability of LRP-1-deficient cells to invade was partially restored when ERK-2 was overexpressed. Since the tyrosine-kinase Src could potentially activate ERK signaling downstream of LRP-1 (Fig. 3), cell invasion was quantified in the presence of a Src kinase inhibitor. However, Src inhibition did not affect the invasive capacities of both shCTRL and shLRP-1 cells (Fig. 4H), thereby excluding Src kinase contribution to LRP-1-mediated ERK activation during carcinoma cell invasion.

### The inhibition of JNK mediated by LRP-1 sustains malignant cell invasion

**Figure 5:**
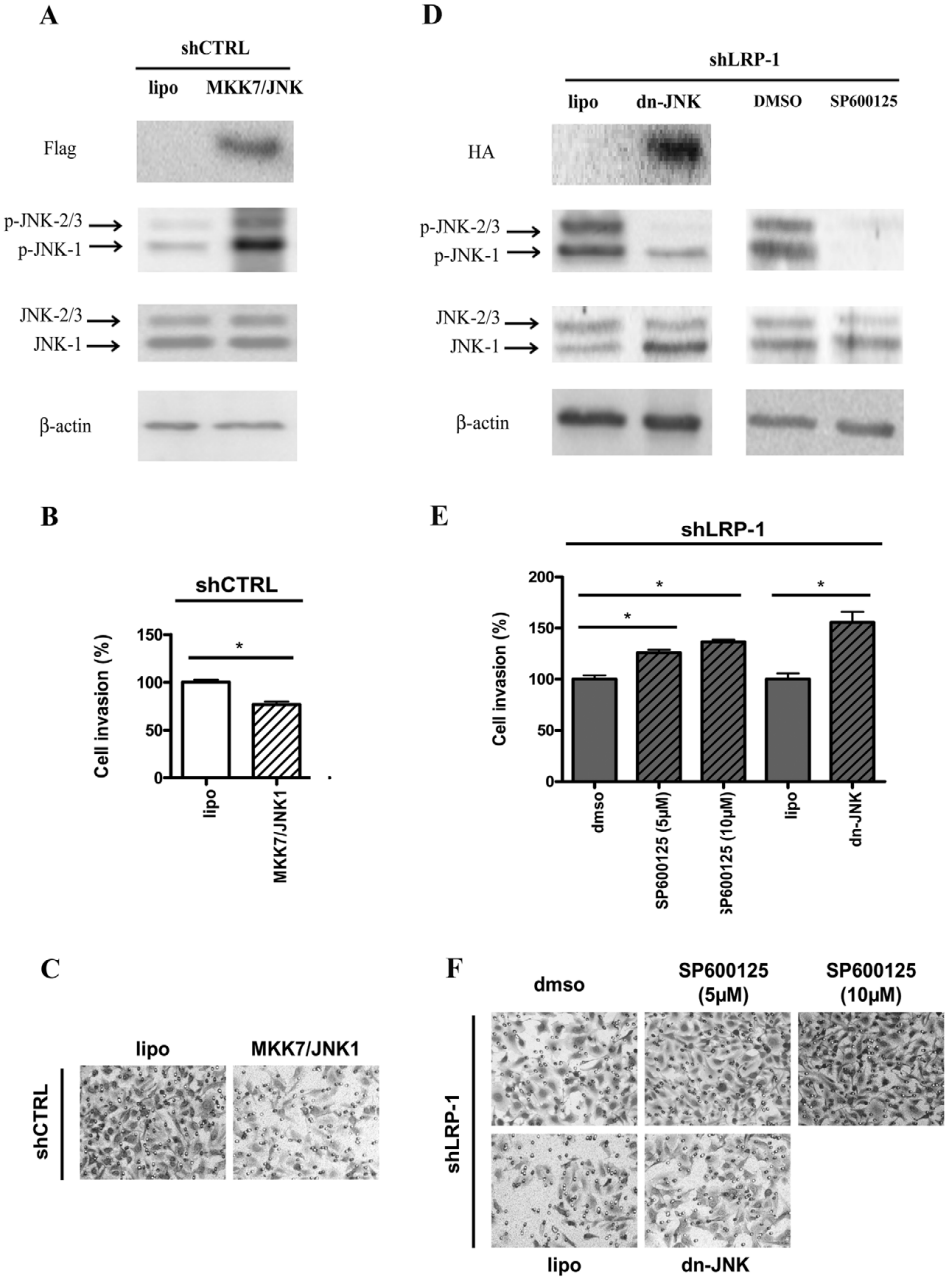
JNK inhibition required for carcinoma cell invasion is mediated by LRP-1. Cell invasion assays were carried out with shCTRL (**B, C**) and shLRP-1 cells (**E, F**) after modulation of JNK activity (**A, D**). (**B, C**) ShCTRL cells were transfected by expression vector coding for wild-type MKK-7/JNK-1 before cell invasion was quantified. (**E, F**) The invasive properties of LRP-1-silenced cells were measured following the SP600125 treatment (5 or 10 µM) or transfection by a dominant-negative mutant of JNK-1 (dn-JNK). JNK activation in shCTRL (**A**) and JNK inhibition in shLRP-1 cells (**D**) was assessed by using phospho-JNK, JNK and β-actin antibodies. Anti-HA and anti-FLAG were used to control the expression of overexpressed tagged proteins. Representative images are shown for each condition (**C, F**). Results were obtained from three separate experiments each performed in triplicate. Invasion was determined by counting cells in eight random microscopic fields per well. Results were expressed as means +/− SD after normalization by comparison with vehicle, i.e., DMSO for drug treatments or lipofectamine (lipo) for transfections. *, *P*<0.05.

Likewise, we examined to what extent LRP-1-mediated inhibition of the JNK pathway could support carcinoma cell invasion. JNK1 and MKK-7 (MAPK kinase-7) were thus co-expressed in shCTRL cells (Fig. 5A). Invasion of shCTRL cells was reduced by 25% when overexpressing wild-type JNK (Fig. 5B and 5C), suggesting that JNK inhibition is required to promote invasion. Next, we used the selective JNK inhibitor SP600125 and a dominant-negative mutant of JNK to recover JNK inhibition in LRP-1-deficient carcinomas (Fig. 5D). We did not observe any significant modulation of ERK phosphorylation under these conditions (Fig. S1). Interestingly, the weak capacity of LRP-1-silenced cells to invade was significantly increased under JNK inhibition (Fig. 5E and 5F). These results support the concept that the inhibition of the JNK-signaling pathway mediated by LRP-1 contributes to carcinoma cell invasion.

### The LRP-1-mediated regulation of MAPK controls the attachment of carcinoma cells

**Figure 6:**
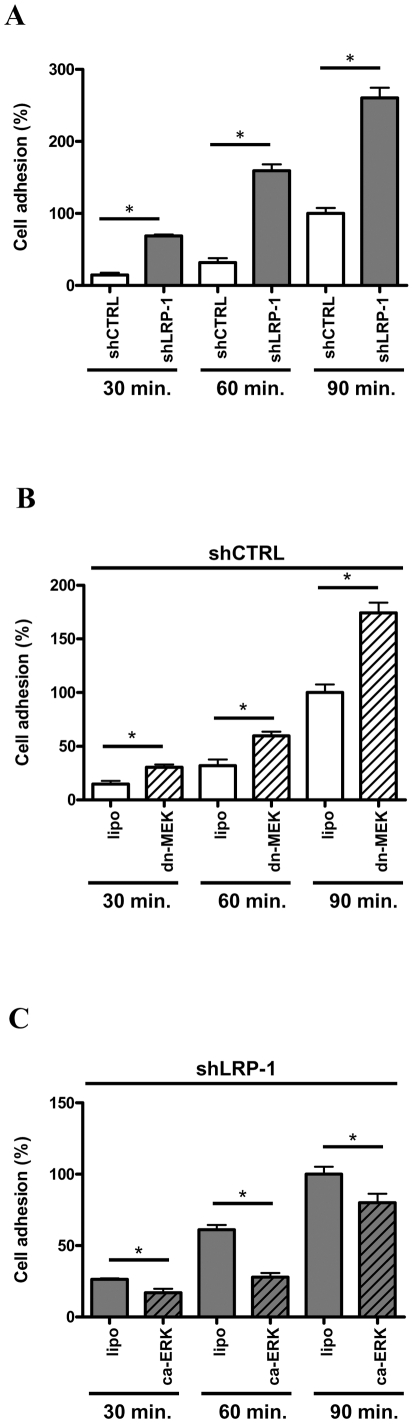
LRP-1-silencing accelerates adhesion of carcinoma cells to matrix substrates through ERK inhibition. (**A**) shCTRL (white boxes) and shLRP-1 (grey boxes) cells were seeded onto gelatin-coated plates and the non adherent cells were discarded after 30, 60 or 90 min. Adhesion assays were also performed with shCTRL cells overexpressing a kinase-dead mutant for MEK-1 (dn-MEK) (**B**) and shLRP-1 overexpressing a constitutively-active ERK-2 (ca-ERK) (**C**). For each condition, results are expressed as percentages of corresponding control adherent cells at 90 minutes. Each value is the mean +/− SD for four separate experiments, with each performed in triplicate. *, *P*<0.05.

**Figure 7:**
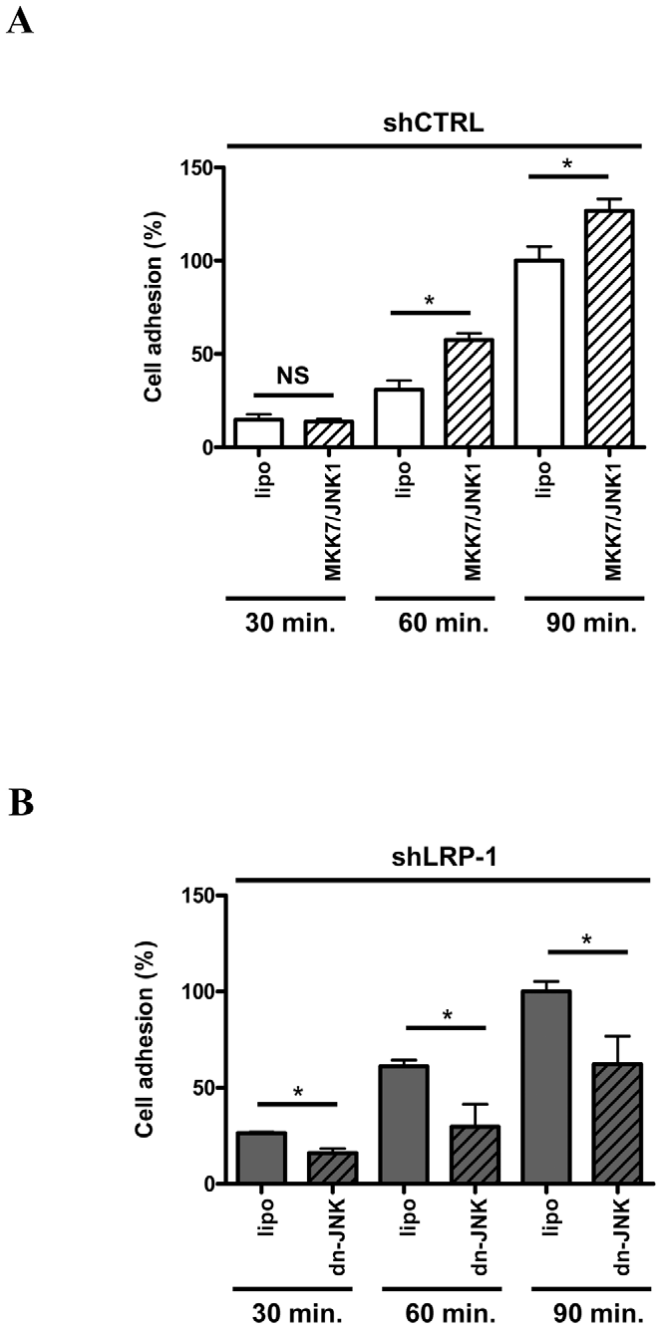
LRP-1-silencing increases adhesion rate of carcinoma cells through JNK hyperactivation. shCTRL (white boxes) and shLRP-1 (grey boxes) cells were seeded onto gelatin-coated plates and the non adherent cells were discarded after 30, 60 or 90 min. Adhesion assays were performed with shCTRL cells overexpressing wild type MKK-7/JNK1 (**A**) and shLRP-1 overexpressing a dominant negative mutant of JNK-1 (dn-JNK) (**B**) kinases. For each condition, results are expressed as percentages of corresponding control adherent cells at 90 minutes. Each value is the mean +/− SD for four separate experiments, with each performed in triplicate. *, *P*<0.05.

We recently reported that LRP-1 silencing severely impaired the invasion of malignant cells consecutively to a strong alteration of cell-matrix interaction turn-over. We therefore postulated that LRP-1-dependent control of both ERK and JNK pathways contributes to modulate the cell-matrix interaction to support invasiveness. As expected, shLRP-1 cells, in which ERK activity is reduced, displayed an accelerated rate of attachment to gelatin-coated surfaces, compared to control clonal cells (Fig. 6A). Moreover, overexpression of a kinase-inactive MEK-1 mutant in shCTRL cells led to increased adhesion rate (Fig. 6B), whereas constitutively active ERK-2 decreased the capacity of LRP-1-deficient cells to attach (Fig. 6C). Indeed, after 60 min of attachment, adherent cells were increased 2-fold upon ERK pathway inhibition (Fig. 6B). At the same time of attachment, the percentage of adhering LRP-1-silenced cells was decreased 2-fold under ERK activation (Fig. 6B and 6C). Likewise, overexpression of wild-type MKK-7/JNK-1 in control cells led to a 2-fold enhanced adherence after 60 min (Fig. 7A). Furthermore, the increased attachment rate observed in LRP-1-deficient cells was reversed by expression of a dominant-negative form of JNK-1 (Fig. 7B). These data support the concept that LRP-1 maintains malignant cells in an intermediate adhesive state that is favorable for invasion through ERK activation and JNK inhibition.

### The actin network of fast-invading carcinomas is rescued in LRP-1-silenced cells following reactivation of ERK or inhibition of hyperactivated JNK

**Figure 8:**
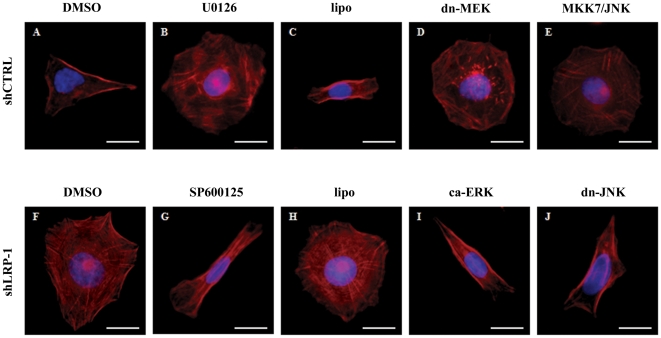
LRP-1 controls carcinoma cells spreading and cytoskeleton organization through MAPK regulation. shCTRL (**A–E**) and shLRP-1 (**F–J**) cells were seeded onto gelatin-coated plates for 120 min. ERK or JNK activities were modulated. Control cells were treated by 25 µM U0126 (**B**) or transfected by a kinase-dead mutant for MEK-1 (dn-MEK) (**D**) to inhibit the ERK-dependent pathway and JNK activation was obtained by overexpression of both wild-type MKK-7 and JNK-1 (**E**). For LRP-1-deficient cells, inhibition of JNK pathway was achieved by using SP600125 treatment (10 µM) (**G**) or overexpression of a dominant-negative form of JNK-1 (**J**), while the reactivation of ERK pathway was obtained by using an expression vector for constitutively-active ERK-2 (**I**). DMSO (**A, F**) served as control for drug treatments (**B, G**) and lipofectamine (**C, H**) served as control for transfection assays (**D, E, I, J**). Cells were stained for actin filaments (red) and nuclei were counterstained with DAPI (blue). Images are representative of at least three separate sets of cultures. Bars, 20 µm.

We further investigated whether the LRP-1-dependent regulation of MAPK signaling could orchestrate the coordination of actin and adhesion dynamics during malignant cell invasion. We therefore analyzed the cellular distribution of fibrillar actin after modulation of ERK and JNK activities (Fig. 8). Adherent control cells displayed a polarized morphology with filopodial cell extensions and a mainly cortically distributed actin network (Fig. 8A). In sharp contrast, LRP-1-silencing induced overspread morphology with numerous stress fibers, prominent transverse filaments and a developed branched actin network (Fig. 8F). Inhibition of ERK signaling in control cancer cells by U0126 treatment (Fig. 8B vs 8A) or a dominant-negative form of MEK-1 (Fig. 8D vs 8C) induced drastic morphological changes similar to those obtained under LRP-1 silencing. The same result was observed after expression of wild type MKK-7/JNK-1 in control carcinoma cells (Fig. 8E vs 8C). In LRP-1-silenced cells, inhibition of JNK signaling by SP600125 (Fig. 8G vs 8F) or overexpression of kinase-inactive JNK-1 (Fig. 8J vs 8H) restored the mesenchymal-like morphology of wild-type carcinoma cells. Moreover, constitutively-active ERK-2 (Fig. 8I vs 8H) was sufficient to reverse the overspread morphology caused by the silencing of LRP-1. These results demonstrated that LRP-1 silencing induces major actin cytoskeleton rearrangements directly linked to ERK inhibition and JNK hyperactivation.

### LRP-1-dependent activation of ERK and inhibition of JNK is necessary for FA disassembly in fast-invading carcinomas

**Figure 9:**
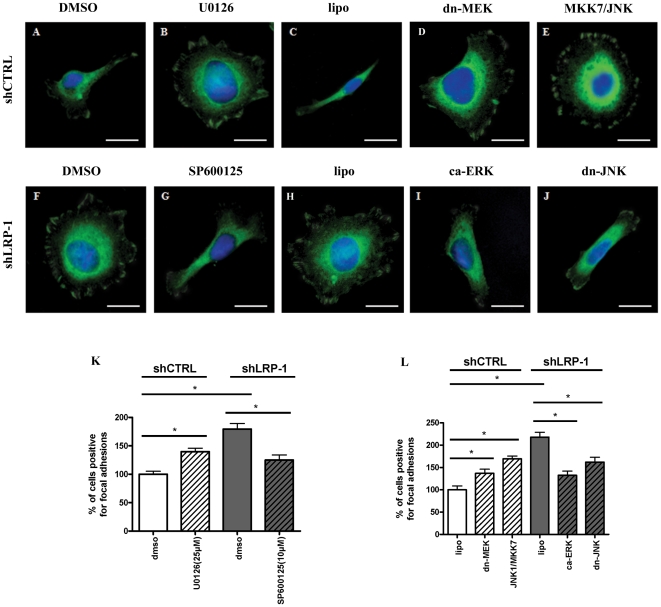
LRP-1 silencing prevents disassembly of focal adhesions through inhibition of ERK and hyperactivation of JNK. (**A–J**) shCTRL (**A–E**) and shLRP-1 (**F–J**) cells were seeded onto gelatin-coated plates for 120 min. ERK signaling pathway was inhibited in shCTRL cells by using 25 µM U0126 (**B**) or a kinase-dead MEK-1 mutant (dn-MEK) (**D**) and reactivated in shLRP-1 cells by a constitutive activation of ERK-2 (ca-ERK) (**I**). The MKK-7/JNK1 vector was used to trigger JNK activation in shCTRL cells (**E**) while JNK inhibition in LRP-1-deficient cells was obtained by using a 10 µM SP600125 (**G**) or a dead-kinase for JNK-1 (dn-JNK) (**J**). DMSO (**A, F**) served as a control for drug treatments (**B, G**) and lipofectamine (**C, H**) served as a control for transfections (**D, E, I, J**). Cells were then stained for talin (green) and nuclei were counterstained with DAPI (blue). Images are representative of at least three separate sets of cultures. Bars, 20 µm. (**K, L**). The percent of cells positive for focal adhesions was quantified following the modulation of ERK and JNK activities by using selective drug treatments (**K**) or the indicated MAPK construct (**L**). For each condition, three hundred cells from three separate experiments were evaluated. Results were expressed in percent, compared to shCTRL cells treated by DMSO (**K**) or lipofectamine (**L**). *, *P*<0.05.

Considering the impact of LRP-1 silencing on the adhesive and morphological properties of carcinoma cells (Figs. 6, 7 and 8), we investigated whether the LRP-1-mediated control of MAPK is necessary for FA turn-over. Thus, the cellular distribution of focal complexes was analyzed in shCTRL and shLRP-1 cells after differential activation of the ERK and JNK pathways (Fig. 9A to 9J). LRP-1-silencing led to accumulation of numerous and highly-structured focal complexes at the cell periphery (Fig. 9F vs 9A). Interference with ERK activation in control cells with U0126 (Fig. 9B vs 9A) or a kinase-inactive form of MEK-1 (Fig. 9D vs 9C) increased the number and size of talin-containing focal contacts to the same extent as observed in LRP-1-silenced cells (Fig. 9F). Accordingly, overexpression of wild-type MKK-7/JNK-1 in LRP-1 expressing control cells stimulated the accumulation of talin-containing peripheral adhesion structures (Fig. 9E vs 9C). By contrast, the number of talin-rich structures was drastically reduced in LRP-1-silenced cells by using a selective JNK inhibitor (Fig. 9G vs 9F) or expression of a dominant-negative JNK-1 mutant (Fig. 9J vs 9H). Similar results were obtained when a constitutively-active ERK-2 was expressed in LRP-1-deficient cells (Fig. 9I vs 9H). These data demonstrated that JNK activation or ERK inhibition in LRP-1-deficient cells could restore the initial distribution of adhesion complexes. The percentage of cells positive for FA was subsequently quantified, as already described [17]. As shown in Fig. 9K and 9L, the number of LRP-1-expressing cancer cells positive for talin-rich adhesion complexes was increased by about 1.4-fold under ERK inhibition (U0126 and dn-MEK) and by 1.7-fold under JNK activation (MKK-7/JNK-1). By contrast, the increased number of focal contacts caused by the LRP-1 silencing was partially reversed by ERK activation (ca-ERK) or JNK inhibition (SP600125 and dn-JNK). Consequently, LRP-1 appears as a main mediator of adhesion disruption through regulation of the ERK and JNK signaling pathways.

### LRP-1 is linked to cytoskeleton and FA components and controls the recruitment of active MAPK to focal complexes

**Figure 10:**
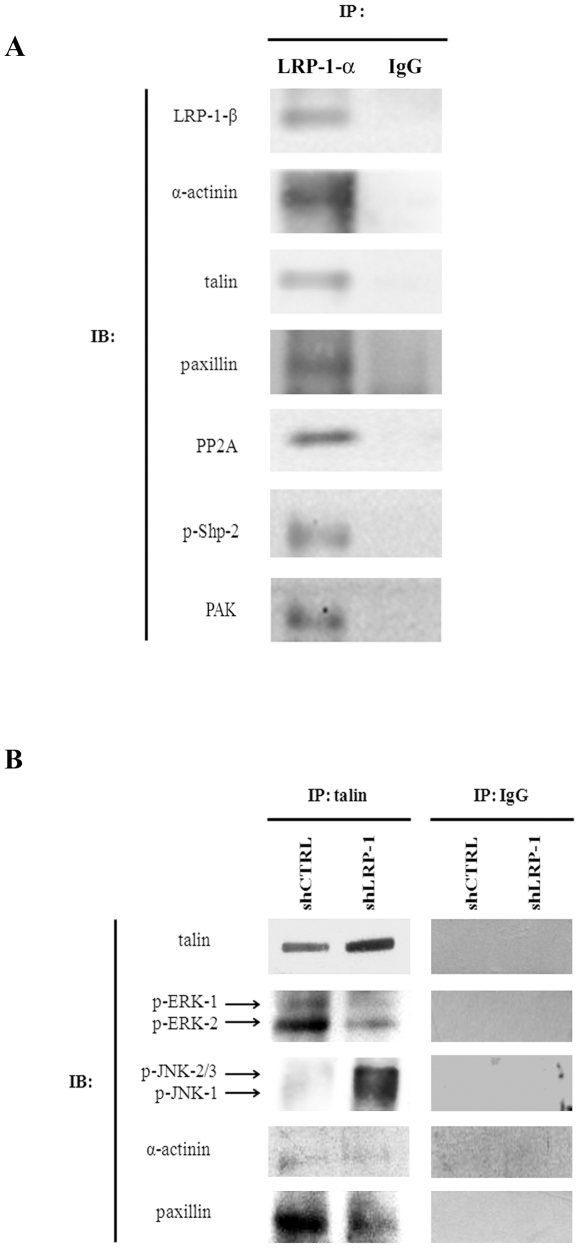
LRP-1 is linked to cytoskeleton and focal adhesion proteins and controls focal contacts composition. shCTRL and shLRP-1 cells were plated on gelatin-coated surfaces and whole-cell extracts were used to perform immunoprecipitation experiments (IP). (**A**) Immunoprecipitation of LRP-1-containing complexes was performed by using the monoclonal anti-LRP-1 antibodies (8G1). Immunocomplexes were then immunoblotted (IB) by using specific antibodies for LRP-1 β-chain, α-actinin, talin, paxillin, phospho-Shp-2, PP2A and PAK. (**B**) Talin-containing complexes were immunoprecipitated and analyzed by Western-blot by using anti-talin, anti-phospho-ERK, anti-phospho-JNK, anti-α-actinin and anti-paxillin antibodies. Nonspecific IgGs were used as a negative control of immunoprecipitation assays.

To clarify the molecular mechanism by which LRP-1 controls the FA dynamics and cytoskeletal organization through MAPK regulation, immunoprecipitation assays were conducted (Fig. 10). The data presented in figure 10A revealed that α-actinin, talin and paxillin interact with the LRP-1 β-chain in fast-invading carcinoma cells. Interestingly, we also detected an association between LRP-1 and active phospho-Shp-2 (SH2 domain-containing protein tyrosine phosphatase-2), PP2A (serine/threonine protein phosphatase 2A), and PAK (p-21 activated kinase) which are key regulators of MAPK signaling. Other molecular relays such as Ras and MEK were also found associated to LRP-1-ICD (Fig. S2). The composition of talin-containing complexes was then analyzed in LRP-1-silenced cells compared to control tumor cells (Fig. 10B). As expected, an increased amount of talin was observed in LRP-1-deficient cells. Moreover, the quantity of paxillin in talin-containing complexes was specifically altered under LRP-1 silencing whereas the α-actinin was not. Interestingly, the active forms of ERK were mainly detected in talin-containing complexes of LRP-1-expressing cells. Furthermore, we observed a strong accumulation of phospho-JNK in these complexes under LRP-1 silencing.

## Discussion

Considering the lack of molecular knowledge about LRP-1 in the cancer field, we investigated the intracellular signaling network connecting LRP-1 to the aggressive behavior of tumor cells. Our results highlighted that LRP-1 maintains malignant cells in an adhesive state favorable for invasion by controlling ERK and JNK-dependent pathways.

We first demonstrated that LRP-1 expression in carcinoma cells triggers the serum-mediated activation of ERK and is responsible for the constitutive inhibition of JNK. Consistent with our results, previous studies have reported a decreased phosphorylation of ERK-1/2 in LRP-1-deficient non-tumor cells [15], [32]. On the other hand, Ma and collaborators reported that LRP-1 could repress ERK activation to control cell mobility [31]. In HT1080 fibrosarcoma cells, Webb and colleagues demonstrated that LRP-1 deficiency led to increased phosphorylated ERK level that stimulates cell migration and invasion [33]. In these studies, activation of ERK in LRP-1-deficient cells required the expression of uPAR, the uPA receptor, which binds vitronectin. These results appeared to be highly vitronectin-dependent since uPAR-vitronectin interaction is well-known to be sufficient to initiate downstream changes in cell migration and signal transduction [34]. The ability of LRP-1 to mediate the endocytic uptake of uPAR and down-regulate the cell surface amount of uPA:uPAR complex was frequently evoked to explain the control of ERK activation by LRP-1 [31], [33], [35]. However, shRNA-mediated knock-down of LRP-1 did not affect the cell surface level of uPAR in our model [17]. Although ERK activation was reported in response to ligand binding to LRP-1 [15], [19], [32], [36], a clear overview of the underlying mechanisms is still lacking. Interestingly, Src-mediated phosphorylation of LRP-1-ICD allows the recruitment of Shc, a scaffold for Ras-ERK activation [37]. More recently, LRP-1 was demonstrated to control ERK activation in neuronal cells through Src-mediated transactivation of Trk (tyrosine kinase receptor) [36]. However, despite the existence of a molecular interaction between Src and LRP-1, a specific Src-inhibitor did not affect the LRP-1-mediated control of cell invasion. This suggests an interesting alternative Src-independent activating platform for ERK. Through immunoprecipitation assays, we detected Shp-2 and PAK association with the LRP-1 β-chain (Fig. 10A). Shp-2 was previously shown to be recruited by LRP-1-ICD and proposed to support ERK signaling during spreading and migration [27], [38], [39]. Additionally, PAK was demonstrated to phosphorylate and activate upstream elements of the ERK pathway [40]. The prospective control of the MKK-7/JNK-1/2/3 pathway by LRP-1 was rarely discussed, although recent studies reported the LRP-1-mediated control of JNK activation in microglial or fibroblastic cells [41], [42]. Cross-talk between JNK and ERK pathways may exist depending on the cell environment. However, a clear contribution of the ERK pathway to JNK signaling was not detected (Fig. S1). Although LRP-1 was recently identified as an activator of the p38 MAPK pathway in brain microvessel endothelial cells [43] and a mediator of the Akt-mediated survival in neurons [44], we did not observe any LRP-1-dependent modulation of these pathways under our experimental conditions (Fig. 1C).

To our knowledge, this is the first report for the existence of interactions between ERK and JNK-containing complexes and full-length LRP-1. Most of the binding partners for LRP-1 were shown to interact with the two NPxY motifs in the cytoplasmic tail [27], [38]. Additional phosphorylation at Ser 73/76/79 and Thr 16 residues were shown to control the tyrosine phosphorylation and probably their accessibility for scaffold molecule recruitment [45]. The stable interactions established by LRP-1-ICD with ERK and JNK may influence the ability of MAPK to reach their downstream targets and may have a repercussion for spatial and temporal propagation of intracellular signaling. Interestingly, MAPK scaffolds, associated with adhesion or growth factor receptors, were reported to persist in active signaling complexes at the surface of endosomal vesicles [46]. Moreover, proteolytically released LRP-1-ICD was recently reported to directly influence transcription through nuclear shuttling [47]. It would be interesting to examine whether LRP-1 would be able to convey ERK or JNK to the nuclear compartment. The presence of JNK on LRP-1-ICD is consistent with studies performed on the Reelin receptor ApoER2 (apolipoprotein E receptor 2)/LRP-8, a close member of the LDL receptor family [48]. Moreover, JNK-interacting proteins JIP-1/2 (JNK-interacting protein-1/2) binding to LRP-1-ICD was demonstrated in yeast two-hybrid and pull-down experiments [28]. In addition, Lutz and collaborators showed that expression of a chimeric construct harboring only the intracellular domain of LRP-1 was sufficient to enhance JNK activation [49]. In this study, the JIP-mediated retention of activated JNK on LRP-1-ICD prevented the nuclear translocation of phospho-JNK, inhibiting the expression of Elk-1 (ets-like protein-1) and c-jun responsive genes. This retention mechanism of active kinases probably did not occur in our carcinoma model since we did not observe phosphorylated forms of JNK bound to LRP-1 (Fig. 3). This could be explained by the ambivalent action of JIP towards JNK. Depending on its ability to recruit various scaffold proteins [50], JIP could also increase the JNK susceptibility to Ser/Thr- and/or Tyr-phosphatase activities associated to the C-terminus of LRP-1 [27], [38], [51]. In agreement, immunoprecipitation assays revealed various protein phosphatases associated with the LRP-1-ICD (Fig. 10A).

Our data demonstrated that LRP-1-mediated regulation of MAPK signaling triggers FA dynamics of cancer cells, supporting cell invasion. LRP-1 emerges as a main regulator of focal complex composition by targeting paxillin, a major scaffold component [52], and phospho-ERK to focal complexes and preventing the localization of JNK in talin-rich structures. Active ERK and JNK were previously identified as FA components in migratory cells [53], [54], [55]. Such a localization was associated with increased phosphorylation of structural and scaffold proteins such as paxillin, triggering the destabilization of FA. Consistent with our results, a recent study reported that uncontrolled JNK-mediated phosphorylation of paxillin results in hypermatured adhesion complexes and impaired migration [56]. Stress fibers, membrane protrusions and projections of fast-invading tumor cells also appeared to be highly regulated by the LRP-1-mediated regulation of ERK/JNK activation. The ability of LRP-1 to target paxillin to FA could control the spatial confinement and activation of Rho family GTPases, well-known to regulate many aspects of actin dynamics [52], [57]. Interestingly, paxillin localization to FA was described as a compelling element of cell polarization through spatial control of Rac activity, a potent regulator of actin branching [58]. In agreement, control of Rac activity by LRP-1 was previously reported in other cell types [31], [49]. Moreover, JNK are downstream effectors of Rac and can induce the activation of WASP (Wiskott-Aldrich syndrome protein) family proteins [59], mediating actin nucleation in membrane ruffles. LRP-1 is likely to support the contractile phenotype of fibroblasts by activating an ERK/Rho/MLCK (myosin light chain kinase) pathway [32] or to stimulate FA disassembly through RhoA inactivation [60]. The presence of LRP-1 at the leading edge [61] could therefore orchestrate polarization and support directional migration of tumor cells. By sensing chemotaxis and haptotaxis gradients, the receptor engages MAPK signaling pathways to control FA composition and cytoskeleton organization and acts as a primary regulator of adhesion disruption. Accordingly, LRP-1 was clearly associated with several aspects of integrin function including their clustering [62], signaling [25] and internalization [10], [63]. Integrin recycling is becoming established as a crucial molecular mechanism that subtly controls cell migration, especially in pathological contexts [64]. The fact that the LRP-1-ICD interacts with various adhesion complex and cytoskeletal components reinforces the close relation it may establish with adhesion receptors and supports its de-adhesive properties towards matrix-engaged integrin complexes. Accordingly, LRP-1- ICD NPxY motifs are shared by integrin-β tails and LRP-1 was shown to bind a talin homologue and the integrin cytoplasmic associated protein ICAP-1 [28], [64].

Finally, by regulating recruitment, activation and targeting of some MAPK to adhesion complexes, LRP-1 contributes to maintain an adhesive state that is favorable for invasion. Recent results reported that LRP-1 may support metastasis development in response to hypoxia [21] or mediate anoikis resistance [23]. As such, LRP-1 may represent a crucial actor in the epithelial-to-mesenchymal transition. Future investigation should focus on identifying MAPK-modulating sequences in LRP-1-ICD to open therapeutic perspectives in the prevention of cancer dissemination.

## Materials and Methods

### Antibodies and chemicals

Anti-LRP-1 α-chain (8G1), anti-LRP-1 β-chain (5A6), anti-human IgGs used as negative control for immunoprecipitation experiments (HP6030), specific SAPK/JNK inhibitor SP600125 (420119) and Src kinase inhibitor I (567805) were obtained from Calbiochem (distributed by VWR International, Strasbourg, France). Anti-ERK-1/2 (9102), anti-SAPK/JNK (9252), anti-Akt (9272), anti-p38 (9212), anti-Src (32G6), anti-phospho-ERK-1/2 (9101), anti-phospho-SAPK/JNK (9251), anti-phospho-Akt (9271), anti-phospho-p38 (9211), HRP-conjugated anti-rabbit (7074) and specific MEK-1/2 inhibitor U0126 (9903) were from Cell Signaling Technology (distributed by Ozyme, Saint Quentin Yvelines, France). Anti-PP2A (1504-1), anti-phospho-Shp-2 (2165-1) and anti-PAK (2247-1) antibodies were purchased from Epitomics (distributed by Euromedex, Mundolsheim, France). HRP-conjugated anti-mouse antibody (NA931V) was from Amersham Biosciences (Buckinghamshire, UK). Anti-talin (MAB1676) was from Chemicon (distributed by Millipore, Molsheim, France). Anti-paxillin (clone 349) antibody was from BD Biosciences (Le Pont de Claix, France). Anti-β-actin (sc-1616) and anti-FLAG (sc-807) antibodies were purchased from Santa-Cruz Biotechnology (distributed by Tebu-Bio, Le Perray en Yvelines, France). Anti-mouse AlexaFluor 488 (A11001), AlexaFluor 568-phalloidin (A12380) and Prolong Gold antifade reagent with DAPI (P36935) were from Molecular Probes (Cergy Pontoise, France). Anti-HA antibody (clone 12CA5) was purchased from Roche Diagnostics (Meylan, France). Anti-α-actinin (BM-75.2), HRP-conjugated anti-goat secondary antibodies (A5420) and other chemicals were from Sigma-Aldrich (Saint Quentin Fallavier, France).

### Plasmids

LRP-1 knockdown was achieved by RNA interference using a previously validated vector-based shRNA approach [17]. Constitutively-active HA-tagged ERK-2 (ca-ERK) and the HA-tagged kinase-dead mutants of MEK-1 (dn-MEK) and JNK-1 (dn-JNK) were generated as described elsewhere [65]. Wild-type MKK-7/JNK-1 vector (FLAG-tagged) was generous gift from Dr. Roger Davis (HHMI, University of Massachusetts Medical School).

### Cell culture and transfection

The human FTC133 cell line was obtained from a lymph node metastasis of a follicular thyroid carcinoma [66]. This cell line was supplied by the European Collection of Cell Cultures, ECACC. Cells were grown in Dulbecco's Modified Eagle Medium-Ham's F12 (Invitrogen, Cergy Pontoise, France) with 10% fetal bovine serum (FBS), as previously described [67]. Generation of two clonal cell lines that stably overexpress a non-silencing sequence (shCTRL) or a specific short hairpin RNA (shRNA) for LRP-1 (shLRP-1) was described elsewhere [17]. For transient transfection assays, plasmids were transfected for four hours by using Lipofectamine 2000 (Invitrogen) according to the manufacturer instructions.

### RNA isolation and RT-PCR

Total RNAs were isolated and purified with an RNeasy isolation kit (Qiagen, Courtaboeuf, France) and RT-PCR (reverse transcription and polymerase chain reaction) was performed as recommended by the manufacturer (Promega, Charbonnières, France). Previously described primers for human LRP-1 [17] and primers for β-actin: forward, GTGTGACGTGGACATCCGC; reverse, CTGCATCCTGTCGGCAATG; were synthesized by Eurogentec (Angers, France). Numbers of cycles were adjusted to ensure that amplifications were in a linear range. The gels shown are representative of at least three separate experiments.

### Western blot analysis

Whole-cell extracts were prepared by scraping cells in ice-cold lysis buffer (25 mM Tris-HCl, pH 7.7, 150 mM NaCl, 0.5% glycerol, 1% Triton X-114, 5 mM EDTA, 0.5 mM EGTA, 1 mM phenylmethylsulfonyl fluoride, 1 mM orthovanadate supplemented with proteinase inhibitor cocktail from Sigma-Aldrich). After 30 min incubation on ice, extracts were centrifugated (10,000× *g* for 10 min at 4°C) and pellets were discarded. The protein concentration in supernatants was quantified by the Bradford method (Bio-Rad Laboratories, Marnes-la-Coquette, France). Proteins were separated by electrophoresis in a 10% sodium dodecyl sulfate-polyacrylamide gel, transferred onto a nitrocellulose membrane (Whatman GmbH, Dassel, Germany), and incubated overnight at 4°C with primary antibodies. Immunoreactive bands were revealed with an ECL plus chemiluminescence kit from Amersham Biosciences by using a ChemiDoc-XRS imaging station from Bio-Rad. Immunoblots presented are representative of at least three separate experiments. The specific signal of β-actin was used to ensure equal loading.

### Immunoprecipitation

The procedure is adapted from [17]. Briefly, for the immunoprecipitation of LRP-1 containing complexes, cell extracts were prepared in the following lysis buffer: 10 mM Tris-HCl, pH 7.4, 150 mM NaCl, 5 mM EDTA, 0.1% Triton X-100, 1 mM phenylmethylsulfonyl fluoride, 1 mM orthovanadate supplemented with proteinase inhibitor cocktail from Sigma-Aldrich. Cell extracts for immunoprecipitation of talin-containing complexes were prepared in a distinct lysis buffer (10 mM Tris-HCl, pH 6.7, 0.75% Brij, 75 mM NaCl, 5 mM EDTA, 1 mM phenylmethylsulfonyl fluoride, 1 mM orthovanadate supplemented with proteinase inhibitor cocktail). After centrifugation (10,000× *g*, 10 min at 4°C) and Bradford titration, 500 µg of proteins were incubated for 4 hours at 4°C on an orbital agitator in presence of anti-LRP-1, anti-talin or nonspecific IgGs. Immunoprecipitation was performed with 40 µL protein G-Sepharose (Amersham Biosciences) for 2 hours at 4°C with constant shaking. The samples were washed three times in the corresponding lysis buffer and protein complexes bound to beads were solubilized under reducing conditions and analyzed by immunoblotting as described above.

### Boyden chamber assay

Matrigel invasion assay was performed using modified Boyden chambers in 24-well dishes with Transwell filter inserts provided with 8-µm pores (Costar, Fisher Scientific Labosi, Elancourt, France), as described elsewhere [67]. Invasiveness was determined by counting cells in eight random microscopic fields per well each seeded in triplicate.

### Adhesion assay

Cancer cell adhesion to gelatin-coated surfaces was measured as previously described [17]. All conditions were assessed in quadruplicate and each presented experiment was performed at least three times.

### Immunofluorescence microscopy

Clonal FTC133 cells were seeded onto gelatin-coated coverslips for 2 hours at 37°C, fixed with 4% paraformaldehyde for 5 min on ice and then permeabilized in ice-cold 0,1% Triton X-100 for 10 min. After three washes with PBS (Phosphate Buffered Saline), cells were incubated 30 min with PBS containing 2% bovine serum albumin to saturate non specific antigens. Coverslips were then incubated for 30 min with AlexaFluor 568-phalloidin or 60 min with anti-talin antibodies followed by three washes with PBS. Talin staining was revealed after 30 min incubation with secondary antibodies conjugated to AlexaFluor 488. Coverslips were then mounted in Prolong Gold antifade medium with 4′,6-diamino-2-phenylindole (DAPI) to obtain nuclei counterstaining. Acquisition was performed with an Olympus BH2-RFCA epifluorescence microscope (Olympus, Japan), equipped with a 100W mercury lamp (OSRAM, GmbH), using a SPlan achromat x 40 objective (Olympus) and fluorescein, rhodamin, DAPI filters for talin, actin and nuclei staining, respectively. Representative images from three separate sets of cultures were treated and merged with ImageJ sofware. The percentage of cells positive for FA was determined as previously reported [17], by examining 300 cells from three different experiments for each condition.

### Densitometric analysis and statistical evaluation

Bands from immunoblots or agarose gels were quantified by using Quantity One image-analyser software (Bio-Rad Laboratories). All culture assays were normalized on the basis of cell viability by using the CellTiter-Glo assay from Promega. Each experiment was performed at least in triplicate and data were expressed as mean +/− SD. Comparisons were performed using Student's t-test (Prism software, GraphPad Inc). Differences from control: NS, not significant; *, *P*<0.05.

## Supporting Information

Figure S1 Analysis of potential cross-talk between ERK and JNK pathways. shCTRL and shLRP-1 cells were plated on gelatin-coated dishes for 24 hours in the presence of FBS. Whole-cell extracts were subjected to immunoblot analysis to study the activation of ERKs (A) and JNKs (B) in the absence or presence of SP600125 (10 µM) and U0126 (25 µM), respectively. Antibodies specific to ERK (A) and JNK (B) kinases were used to ensure equal loading. The immunoblots presented are representative of at least three separate experiments.
(0.41 MB TIF)

Figure S2 Ras and MEK are co-immunoprecipitated with LRP-1-in tumor cells. Cell lysates from shCTRL cells were subjected to immunoprecipitation assays using anti-LRP-1 alpha-chain antibodies (IP: LRP-1). The immunocomplexes were then immunblotted (IB) with anti-LRP-1 beta-chain (5A6), anti-Ras (2006992, Millipore), anti-Raf (sc-166, Santa-Cruz) and anti-MEK-1/2 (sc-436, Santa-Cruz) antibodies. Nonspecific IgGs were used as the negative control for immunoprecipitation.
(0.28 MB TIF)
