# Supplementary figures and images for: LRP-1 Promotes Cancer Cell Invasion by Supporting ERK and Inhibiting JNK Signaling Pathways
Source: PLoS One. 2010 Jul 14;5(7):e11584. doi: 10.1371/journal.pone.0011584 (PMC2904376; doi:10.1371/journal.pone.0011584)

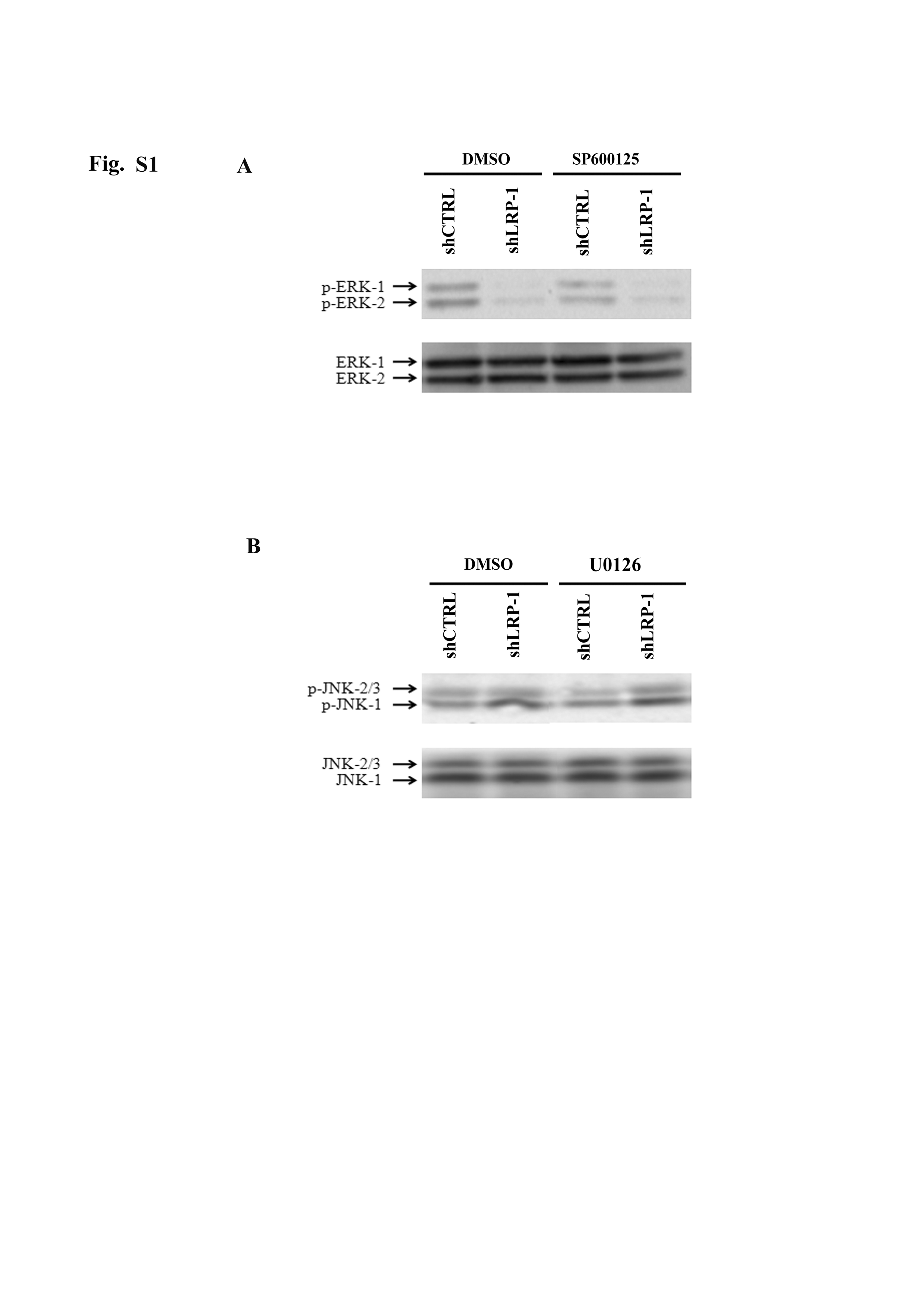

Supplement: Figure S1 — Analysis of potential cross-talk between ERK and JNK pathways. shCTRL and shLRP-1 cells were plated on gelatin-coated dishes for 24 hours in the presence of FBS. Whole-cell extracts were subjected to immunoblot analysis to study the activation of ERKs (A) and JNKs (B) in the absence or presence of SP600125 (10 µM) and U0126 (25 µM), respectively. Antibodies specific to ERK (A) and JNK (B) kinases were used to ensure equal loading. The immunoblots presented are representative of at least three separate experiments. (0.41 MB TIF) [file pone.0011584.s001.tif]

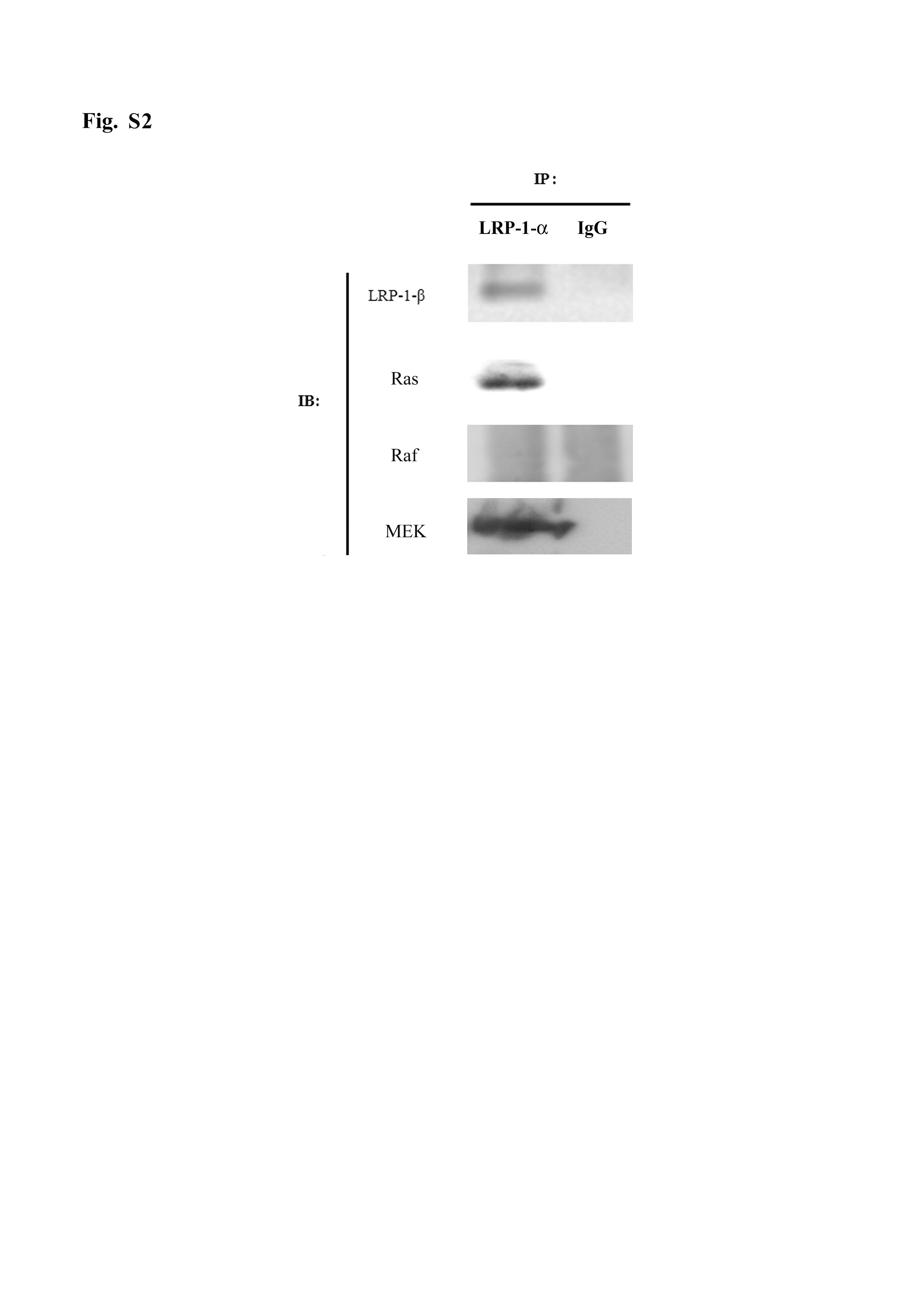

Supplement: Figure S2 — Ras and MEK are co-immunoprecipitated with LRP-1-in tumor cells. Cell lysates from shCTRL cells were subjected to immunoprecipitation assays using anti-LRP-1 alpha-chain antibodies (IP: LRP-1). The immunocomplexes were then immunblotted (IB) with anti-LRP-1 beta-chain (5A6), anti-Ras (2006992, Millipore), anti-Raf (sc-166, Santa-Cruz) and anti-MEK-1/2 (sc-436, Santa-Cruz) antibodies. Nonspecific IgGs were used as the negative control for immunoprecipitation. (0.28 MB TIF) [file pone.0011584.s002.tif]
